# The Role of Air Adsorption in Inverted Ultrathin Black Phosphorus Field-Effect Transistors

**DOI:** 10.1186/s11671-016-1737-6

**Published:** 2016-11-25

**Authors:** Qianqian Li, Jiancui Chen, Zhihong Feng, Liefeng Feng, Dongsheng Yao, Shupeng Wang

**Affiliations:** 1 Tianjin Key Laboratory of Low Dimensional Materials Physics and Preparing Technology, School of Science, Tianjin University, Tianjin, 300072 China; 2 State Key Laboratory of Precision Measuring Technology & Instruments, College of Precision Instrument and Opto-electronics Engineering, Tianjin University, Tianjin, 300072 China

**Keywords:** Inverted structure, Field-effect transistors (FET), Annealing, Air adsorption

## Abstract

Few-layer black phosphorus (BP) attracts much attention owing to its high mobility and thickness-tunable band gap; however, compared with the commonly studied transition metal dichalcogenides (TMDCs), BP has the unfavorable property of degrading in ambient conditions. Here, we propose an inverted dual gates structure of ultrathin BP FET to research the air adsorption on BP. In fabrication process of back-gate BP FET, BP was transferred directly onto a wafer covered with electrodes. Thus, we can exclude the BP degradation during the process of electrodes fabrication, such as electron beam lithography (EBL) and thermal evaporation process. Furthermore, without any electrode covering BP, BP could be in full contact with the air; then the accurate effect of the air adsorption on BP can be researched in detail. The results clearly show that annealing can remove the *p*-doping resulted from the metastable oxygen adsorbed on the surface of BP, but the adsorption can be restored in a few hours exposure. In addition, both back and top gate inverted BP FETs exhibit a favorable performance. Therefore, this inverted structure is also an optional structure to reduce the influence of the instability of BP devices.

## Background

Phosphorus is the second discovered monotypic Van der Waals two-dimensional (2D) material. Its bandgap achieves both larger on/off ratios than in graphene transistors and higher field-effect mobility compared with TMDCs such as MoSe_2._ It was found to be naturally *p*-type with a direct bandgap of 0.3 eV, which was predicted to increase to approximate 2.0 eV in a monolayer remaining direct bandgap [1, 2]. Therefore, it is suitable for transistor, logic, and optoelectronic applications. Despite the good points of BP, a major challenge for its practical application is the instability in the ambient environment. It gets degradation upon exposure to ambient air [3, 4]. Previous studies suggested that the performance of degradation upon exposure to the air is mainly attributed to the adsorption of water or oxygen on the material surfaces and the chemical reactions [5], and this adsorption of few-layer BP is generally supposed to lead to *p*-doping upon exposure to air [6–8].

In order to get the detailed effect of the adsorption on BP, we propose an inverted structure of BP FET. In fabrication process of back-gate BP FET, the BP was transferred directly onto a wafer covered with electrodes. In traditional FET, the electrodes are fabricated on BP directly. Compared with it, the inverted few layers back-gate BP FET has two advantages to getting the role of air adsorption on BP. Firstly, the degradation of BP during the EBL and the thermal evaporation process could be eliminated. Secondly, it can ensure sufficient contact between the air condition and BP because the effect of electrodes covering on BP is avoided. For example, if the device had been fabricated into the traditional structure in our fabrication process, the electrodes should have covered more than half effect area of BP. Both advantages ensure that we could clearly observe the change process of the dominant carrier type. Results provide a conclusive proof that annealing can remove the *p*-doping that resulted from the metastable oxygen adsorption on the surface of BP [9, 10]. In addition, both this inverted back- and top-gate BP FETs exhibit favorable mobility. Therefore, this inverted structure could be an optional structure to reduce the influence of the instability of BP devices.

## Methods

**Fig. 1:**
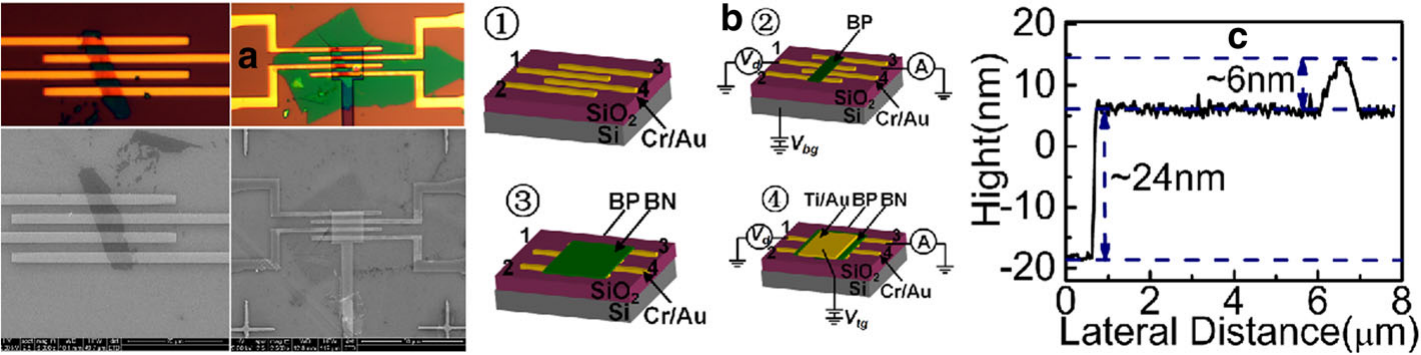
**a** Optical image and SEM image of the inverted FET. **b** Fabrication process of the device. ① The first EBL patterning obtain the Cr/Au electrode (15/60 nm), the channel of each electrode is 2 μm. ② Transfer of the ultrathin BP. ③ Transfer of the *h*-BN. ④ The second EBL patterning obtain the Ti/Au electrode (20/60 nm). **c** The AFM integrated data, BP thickness of 6 nm on the top of 24 nm *h*-BN

The fabrication process is illustrated in Fig. 1b. Metal contacts were deposited on degenerately doped silicon wafer covered with a layer of thermally grown silicon dioxide by EBL and thermal evaporation of chromium and gold (typically 15 and 60 nm, respectively), then a scotch type-based mechanical exfoliation method was used to peel a few layers of BP from bulk crystal onto silicon wafer-fabricated polyvinyl alcohol (PVA) and polymethyl methacrylate (PMMA). Then BP was transferred onto a wafer covered with electrodes already. The PMMA was removed by overnight treatment with acetone and then 15 min treatment with IPA [11]. After annealing at 200 °C for 3 min, we measured the transport characteristic before and after annealing of the back-gate devices. We passivated the BP surface with *h*-BN which has been demonstrated to protect the devices from degrading in the ambient environment for 1 week [12], the transfer method of *h*-BN is the same as BP, followed by a second EBL and thermal evaporation process to obtain the top-gate electrode. Optical microscopy and atomic force microscopy (AFM) were used to find few layer samples and to determine their thickness (Fig. 1a, c). The surface roughness of BP was verified using SEM before and after fabrication (Fig. 1a). In traditional 2D material FET structure, the ultrathin BP is transferred before EBL patterning, which could contribute to momentary exposure in ambient conditions and thereby cause slightly degraded performance. In this inverted structure, the BP back-gate FET device did not go through the step of EBL patterning, metal deposition, and lift-off process.

## Results and Discussion

**Fig. 2:**
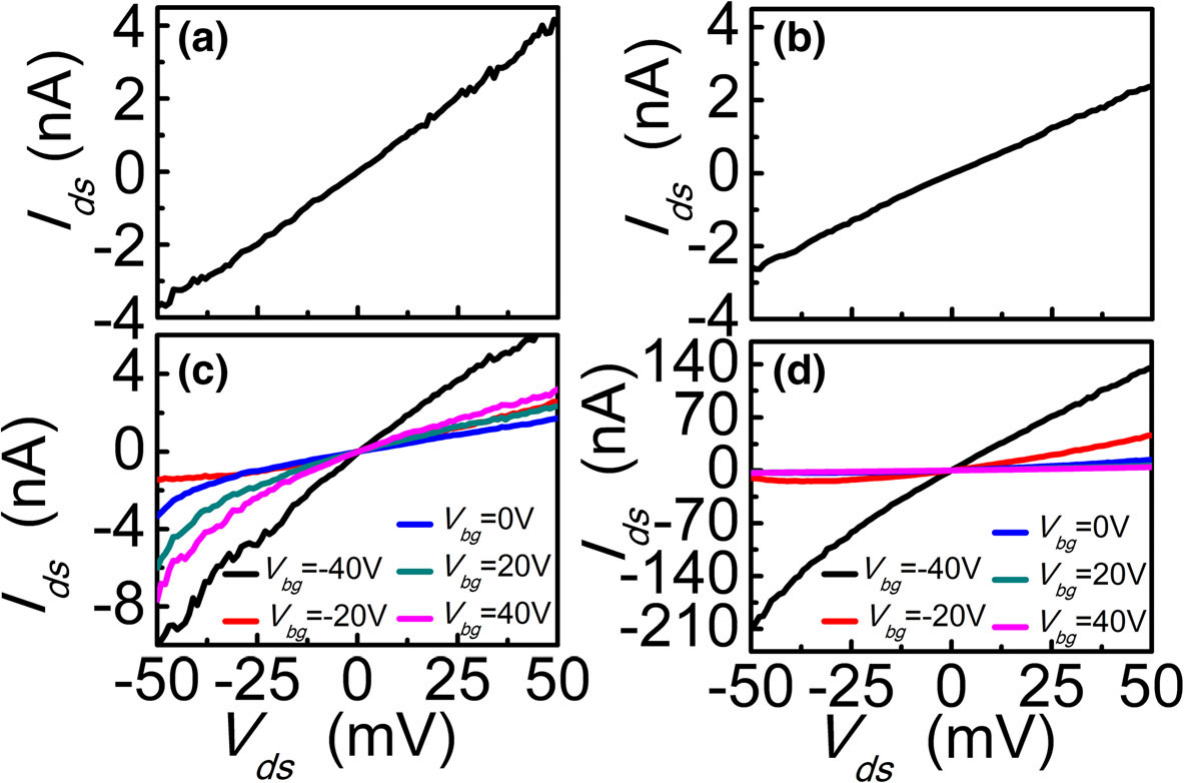
**a** *I*-*V* characteristic without annealing as no gate swept. **b** *I*-*V* characteristic just after annealing as no-gate swept. **c** Output characteristic for back-gate swept from −40 to 40 V just after annealing. **d** Output characteristic for back-gate inverted BP FET swept from −40 to 40 V after annealing about 5 h

Figure 2a, b show the drain current-voltage curves of the inverted FET without annealing and just after annealing as no gate swept, at a small drain-to-source voltage (*V*_*ds*_) of 50 mV, respectively. Both the drain currents vary linearly in this region with the small *V*_*ds*_. However, there is no significant increase in the source current of the inverted FET just after annealing, as shown in Fig. 2b, which goes contrary to the theory that annealing will lead to a better contact [13]. Under various back-gate biases (−40, −20, 0, 20, and 40 V), the output characteristics of the inverted device just after annealing and after annealing about 5 h with the small *V*_*ds*_ of 50 mV are shown in Fig. 2c, d, respectively. Compared with just after annealing, the source current after annealing about 5 h significantly increases.

**Fig. 3:**
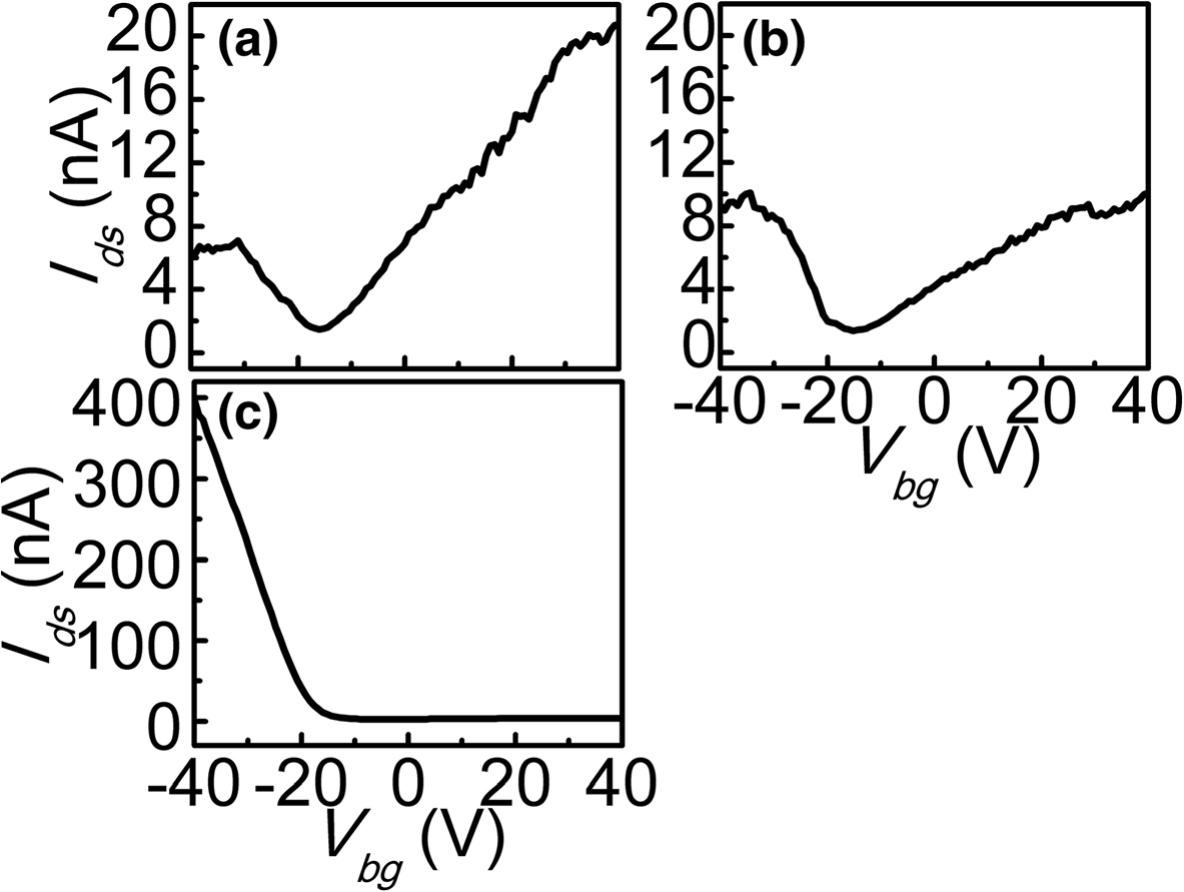
**a** Transfer characteristic of back-gate inverted BP FET after annealing about 5 min, *p*-doping is weaker than *n*-doping. **b** Transfer characteristic of back-gate inverted BP FET after annealing about 10 min, *p*-doping enhances slowly while *n*-doping fades away. **c** Transfer characteristic of back-gate inverted BP FET after annealing about 5 h, *n*-doping reduces to near zero

In order to explain the above experiments that *I*_*ds*_ does not increase just after annealing but sharply increases after annealing about 5 hours, we measured the transfer characteristics under these two conditions, as shown in Fig. 3. Theoretical and experimental studies have shown that metastable oxygen adsorbed on the surface of few-layer BP will lead to *p*-doping upon exposure to air, but the adsorption can be removed during the annealing [13, 14], then leading to a reduction in *p*-doping. In Fig. 3a, the absorption should be removed just after annealing, then the dominant carrier type in BP changes from naturally *p*-type to *n*-doping. With the increasing of exposure time, re-adsorption of the metastable oxygen on BP surface leads to *p*-doping enhancing slowly while *n*-doping fading away, as shown in Fig. 3b. After enough exposure time, the *n*-doping of the device reduces to near zero, the dominant carrier type changes to *p*-doping again, as shown in Fig. 3c.

Normally, the high work function of the metal electrodes such as Cr/Au we used will cause hole accumulation at the metal-semiconductor interface, which forms a low-resistance ohmic contact for the *p*-doping device; while for the *n*-doping device a depletion region is formed at the interface, which leads to Schottky barriers [15, 16]. According to this theory, in our devices the dominant carrier type changes to *n*-type just after annealing, then Schottky barriers are formed and consequently a small current; however, with the re-adsorption of the metastable oxygen on the BP surface, the *p*-type recovers, which matches well with the high work function metal electrodes as we used. At the same time, annealing will result in better contact, so the current sharply increases after annealing about 5 h. In summary, according to the results of Fig. 3 we can conclude that the work function mismatch between the metal contacts and few-layer BP should be the main reason that the current does not increase in Fig. 2b, c but significantly increases in Fig. 2d.

**Fig. 4:**
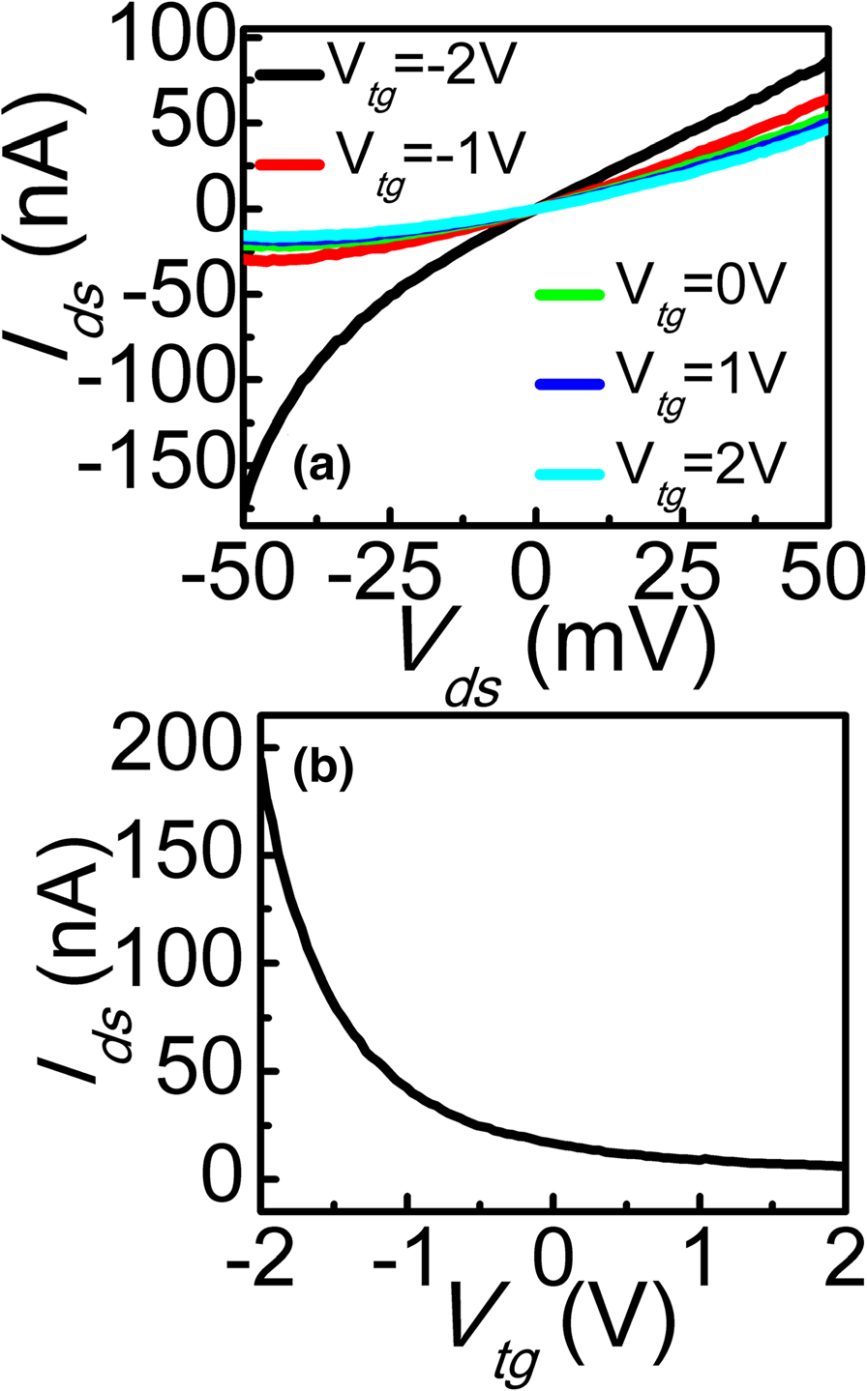
**a** Output characteristic for top-gate inverted BP FET swept from −2 to 2 V. **b** Transfer characteristic of top-gate inverted BP FET

Previous reports have proved that the BP degradation process can be slowed down considerably by covering the material with appropriate passivation layers, and it is stable for more than a week [17, 18]. Here we passivated the device with *h*-BN and then fabricated a top-gate. Figure 4a, b show the output and transfer characteristics of the device. Obviously nonlinear behavior of *I*_*ds*_ varying with different *V*_*ds*_ indicates the formation of Schottky barrier at the contacts. It may be caused by the residue of PMMA. On the other hand, residue could increase the thickness of the dielectric layer, which could weaken the effective control of the channel carrier concentration by the gate electrode [19]. Therefore, if the gate is not adequately strong, the drain will decrease the gate over the channel control, resulting in poor-gate dependence shown in Fig. 4b compared with Fig. 3c. In addition, both back- and top-gates devices exhibit a respectable on/off ratio of 10^2^ with small *V*_*ds*_ of 0.05 V. In our measurement, due to the doping level limited by the breakdown electric field of the gate dielectric, the on-state current of our devices does not yet reached saturation. It is therefore possible to achieve even higher drain-current modulation by using high-*k* materials as gate dielectrics for higher doping [20, 21].

The field-effect mobility of devices was exacted from the linear scales shown in Figs. 3c and 4b using the expression:

1 $$ {\mu}_{FE}=\frac{d{I}_{ds}}{d{V}_g}\frac{L}{W{C}_i{V}_{ds}} $$

where *L*/*W* is channel length/width ratio, in our experiments *L* is 6 μm, *W* is about 5 μm, then the ratio is 1.2. *C*_*i*_ = *ε*_*0*_ *ε*/*d* is the capacitance per unit area [5], *ε*_*0*=_8.854 × 10^-12^fm^-1^ is the permittivity of vacuum, *ε =* 3.9 is the relative dielectric constant of SiO_2_, and the relative dielectric constant of *h*-BN is ranging from 3 to 4 [22]. *d* is the thickness of the dielectric layer, for SiO_2_ is 300 nm, and for *h*-BN is 24 nm. By using the maximum slope of the *I*_*ds*_-*V*_*g*_ plots, at *V*_*ds*_ = 0.05 V, a field mobility for back-gate sweeps and top-gate sweeps are, respectively, 50 cm^2^v^-1^s^-1^ and 75 cm^2^v^-1^s^-1^ in our measurement range. From *S* = *dV*_*bg*_/*d*log*I*_*sd*_, we can obtain an excellent subthreshold swing *S* ≈ 1.3 V/*dec* of top-gate FET on *h*-BN compared with *S* ≈ 6.8 V/*dec* of back-gate FET on SiO_2._

## Conclusions

In summary, we prepared an inverted ultrathin BP FET to get the role of air adsorption on BP. In fabrication process of back-gate BP-FET, the BP was transferred directly onto a wafer covered with electrodes. In traditional FET, the electrodes are fabricated on BP directly. Compared with it, our inverted back-gate BP FET could keep BP complete contact with air condition and does not need to consider BP degradation during BEL and thermal evaporation process as well as the cover effect of electrodes on BP. Therefore, the effects of annealing and exposure time after annealing on the electrical behaviors of ultrathin BP back-gate FET could be measured accurately. Just after annealing the adsorption is removed, then the dominant carrier type in BP changes from naturally *p*-type to *n*-doping. With the increasing of exposure time, re-adsorption of the metastable oxygen on BP surface leads to *p*-doping enhancing slowly while *n*-doping fading away. After enough exposure time, the *n*-doping of the device reduces to near zero, the dominant carrier type changes to *p*-doping again. In addition, top-gate ultrathin *h*-BN BP FET also exhibits favorable mobility and lower subthreshold swing compared with back-gate FET. On the whole, both back- and top-gate ultrathin inverted BP FETs exhibit high performance. Therefore, this inverted structure is an optional structure to reduce influence of the instability of BP devices because of two potential advantages, one is to avoid the time of BP exposure to air as long as it is timely covered by *h*-BN, and the other is to reduce an electrode preparation after BP transfer.

## Abbreviations

2D: Two-dimensional
AFM: Atomic force microscopy
BP: Black phosphorus
EBL: Electron beam lithography
*V*_*ds*_: Drain-to-source voltage
